# Rehabilitation interventions for glioma patients: a mini-review

**DOI:** 10.3389/fsurg.2023.1137516

**Published:** 2023-06-16

**Authors:** Stefania Spina, Salvatore Facciorusso, Nicoletta Cinone, Raffaello Pellegrino, Pietro Fiore, Andrea Santamato

**Affiliations:** ^1^ Department of Medical and Surgical Sciences, Spasticity and Movement Disorders “ReSTaRt”, Unit Physical Medicine and Rehabilitation Section, University of Foggia, Foggia, Italy; ^2^ Department of Medical and Surgical Specialties and Dentistry, University of Campania “Luigi Vanvitelli”, Naples, Italy; ^3^ Department of Scientific Research, Campus Ludes, Off-Campus Semmelweis University, Lugano, Switzerland; ^4^ Neurorehabilitation Unit, Istituti Clinici Scientifici Maugeri, IRCCS, Institute of Bari, Bari, Italy

**Keywords:** glioma, brain tumor, rehabilitative care, physical therapy, cognitive theraphy, rehabilitation interventions

## Abstract

Glioma is a group of tumors that originate from glial cells within the central nervous system and comprise 27% of all tumors and 80% of malignant tumors. With remarkable progress in surgical practices, chemotherapy, and radiation therapy, patients with glioma are experiencing greater survival times, which means they need more rehabilitative care. In fact, people with this condition may experience a variety of symptoms that can affect their functions and drastically reduce their quality of life. In fact, patients suffering from glioma has a distinctive symptom complex highlighting the requirement for customized care. Growing evidence shows that rehabilitation therapy can improve the functional prognosis and quality of life of glioma patients. However, there is limited evidence of the success of rehabilitation protocols designed specifically for individuals with glioma. It is essential to determine the most comprehensive rehabilitation programs as well as the sufficient resources, dosage, and duration. The goal of this mini-review was to classify and map rehabilitation interventions used to treat multiple disabling sequalae in individuals affected by glioma. We aim to provide a comprehensive overview of the rehabilitation protocols used for this population, so that clinicians have a guide to support treatment and an inspiration for further research. This document is intended to be a reference point for professionals involved in the management of adult patients with gliomas. Further exploration is needed to form improved care models for recognizing and addressing functional restrictions in this population.

## 1. Introduction

Gliomas are tumors that originate from glial cells in the central nervous system. They can be divided into two main groups: diffuse gliomas, which infiltrate their surroundings, and those with a more circumscribed growth pattern (1). The World Health Organization updated the Classification of Tumors of the Central Nervous System in 2021 (2). This update separated paediatric-type and adult-type diffuse gliomas, as they differ in clinical and biological aspects (3). Recent advances in surgical techniques, chemotherapy, and radiation therapy have increased the survival of glioma patients, but they also need more rehabilitation support and services (4).

Patients diagnosed with glioma have specific functional impairments and restrictions in physical activity, making them a distinct patient population. They experience many symptoms that affect their neurological functions and quality of life. A recent review (5) reveals that the ten most prevalent symptoms in glioma patients are: seizures (37%), cognitive deficits (36%), drowsiness (35%), dysphagia (30%), headache (27%), confusion (27%), aphasia (24%), motor deficits (21%), fatigue (20%) and dyspnea (20%). Moreover, approximately 60% of glioma patients are insufficiently active or sedentary and do not meet the Physical Activity Guideline provided by the World Health Organization (6). Glioma patients are a distinct group within the cancer population because of their unique burden of symptoms. The International Classification of Functioning, Disability and Health (ICF) framework can help to explain how this disease impacts different areas of life, such as impairments (headaches, seizures, etc.), activity (decreased mobility), and participation (work, family, etc.), affecting the quality of life (7).

The purpose of rehabilitation is to effectively improve the motor, consciousness and psychology of glioma patients (8). The guidelines stated that patients “with any type of brain tumor should be referred for rehabilitation consultation upon diagnosis and at every stage of following up, including with metastatic disease” (9). However, crucial information on best practices, settings, type, intensity, duration, and cost-effectiveness of therapy is lacking.

To accurately assess the issues and plan effective interventions, a range of factors needed to be considered and only a multidisciplinary approach could ensure that each person can receive support tailored to their individual physical, sensory, cognitive, psychological and social needs. Preliminary research suggests that rehabilitation can reduce disability in glioma patients, by improving both functional abilities and cognitive functioning compared to standard care. A recent meta-analysis (10) indicates that patients with glioma receiving rehabilitation have better outcomes on neurological function and quality of life.

To date, there is limited evidence of the success of rehabilitation programs designed specifically for individuals with glioma. The impact of multidisciplinary treatment was examined by a Cochrane Review completed in 2013, although no RCTs could be included due to the difficulties in locating high-quality research. The twelve lower-quality observational studies included led the authors to draw the following conclusions: interdisciplinary rehabilitation is not detrimental and may even improve disability and quality of life (11). A few years later, the same authors provided only low evidence that multidisciplinary rehabilitation may reduce short- and long-term motor disabilities in people after primary brain tumor treatment. Indeed, there is still a gap in this research area and the need for new research is evident (11).

This mini-review is not intended to be a comprehensive review of the available literature on glioma tumor rehabilitation as it serves as a guide for clinicians to support treatment and facilitate further research. We aim to offer a comprehensive overview of the rehabilitation protocols used for this population.

## 2. Rehabilitation intervention

### 2.1. Literature research

References for this review were obtained by searching the online databases PUBMED, CINAHL, Web of Science, Cochrane, and Scopus until November 2022. Search terms included: brain, glioma, rehabilitation, intervention with the Boolean operator “AND” to narrow results and connect terms. We considered articles published after the year 2000 and papers printed in or translated into English. Only observational studies and randomized control trials were included. Some of the references were not found through online databases, but rather through reference lists of other articles.

The raw search of the databases yielded a total of 164 articles, then an initial filtering within the same databases by document type (Article; Clinical Trial;) and a subsequent filtering of duplicate articles, left 31 unique articles. These 31 articles were subjected to a review of titles and abstracts, leaving 29 articles as candidates for assessment for eligibility. Only 13 articles met the inclusion criteria with 16 articles being excluded. To ensure the homogeneity of the study population, we excluded 6 papers that reported on various brain tumor types, such as meningiomas, metastases, and other tumors, besides glioma (Prisma Flow Chart in Supplementary Material).

Studies included in the present review are those reporting a full description of the rehabilitation protocol applied. For this research, we used the TIDieR (Template for Intervention Description and Replication) checklist to capture intervention descriptions. This checklist included categories such as “brief name”, “why”, “what (materials)”, “what (procedures)”, “who provided”, “how”, “where”, “when and how much”. The TIDieR checklist is designed to ensure that authors provide enough detail for others to replicate their interventions.

Data extracted are summarized in Tables 1, 2. Studies are divided in two groups: physical and cognitive therapy.

**Table 1:** Characteristics of the included studies.

Reference	Study design	Diagnosis	Sample size	Age (years) mean ± SD	Rehabilitative Intervention	Evaluation timing	Outcomes	Main Results
Physical Rehabilitation								
Hansen et al. 2017	OS	Glioma (WHO grade I–IV)	24	median: 62 interval: 20–77	IG: PT and OT	T0: baseline T1: end of treatment	EORTC QLQ-C30; EORTC-QLQ-BN20; BBS; 10 mWT; Watt-Max cycle test; AMPS	Significant improvement in HRQOL and symptom outcomes in terms of EORTC-QLQ-30 “emotional function”, symptom item “fatigue” and EORTC-QLQ-BN20 symptom scale “future uncertainty”. Significant improvement in strength of leg press by 32%, knee extension by 17%, knee flexion by 15%, arm flexion by 25%, and arm extension by 26%, and reduced the step frequency for walking 10 meter.
Ghering et al. 2018	RCT	Glioma (WHO grade II–III)	34 (23 IG; 11 CG)	IG: 48.0 ± 9.4; CG: 48.0 ± 11.9	IG: Individual home-based exercise; CG: care	T0: baseline T1: end of treatment (6 mo)	Absolute and relative VO2 peak; International Physical Activity Questionnaire (IPAQ)	Mean absolute VO2 peak in the exercise group improved 6.0% compared to baseline, and mean relative VO2 peak improved 7.3%. The median self-reported physical activity increased in both groups, but more strongly in the exercise group (126% in the exercise group compared to 23% in the control group).
Yu et al. 2019	OS	Glioma (WHO grade I–IV)	35	57.6 ± 13.2	IG: PT and OT	T0: baseline T1: 1mo after	FMA; BBS; MBI; MMSE; IQ; ECOG	Significant improvement in FMA, BBS, MBI, MMSE, and IQ scores (*p* < 0.001) and within benign and malignant brain tumor subgroups (*p* < 0.05).
Hansen et al. 2020	RCT	Glioma (WHO grade II–IV)	64 (32 IG; 32 CG)	IG: 56.1 ±: 11.6 CG: 52.1 ±: 13.4	IG: PT and OT CG: standard care	T0: baseline T1: 6 weeks	EORTC-QLQ-30; GHS/QoL; submaximal Åstrand-Rhyming Cycle Ergometer Test.	Nonsignificant increase in the overall QoL score. The IG had a significantly better score on the HRQoL domain cognitive functioning, significantly reduced symptoms of fatigue, visual disorder, communication deficits, headaches, drowsiness, and itchy skin; and significantly better scores on functional performance outcomes, including aerobic power, muscle strength of leg press, elbow extension, and elbow flexion.
Tay et al. 2022	OS	Glioma (WHO grade I–IV)	163	55.5 ± 13.3	IG: PT and OT	T0: baseline T1: discarge	FIM	Mean gain in motor FIM of 18.8 ± 12.9 and a mean gain in cognitive FIM of 3.85 ± 6.31 from admission to discharge.
Cognitive Rehabilitation								
Gehring et al. 2009	RCT	Glioma (WHO grade II–III)	140 (70 IG; 70 CG)	IG: 49.2 ± 8.9 CG: 48.0 ± 11.9	IG: cognitive training CG: waiting listing + motivational brochure	T0: baseline T1: end of treatment (6 weeks)	Stroop Color-Word Test; Digit Span; Letter Digit Substitution Test; Memory Scanning Test; TEA; Elevator Counting with Distraction; Visual Verbal Learning Test; Concept Shifting Test; Letter Fluency; Category Fluency; BADS; Elevator Counting with Reversal; Telephone Search While Counting	Signiﬁcant improvement in self-reported cognitive functioning at the immediate post intervention assessment (*p* < 0.001), but not at the 6-month follow-up. Conversely, although no signiﬁcant group differences in neuropsychological test scores were observed at the immediate postintervention, clear differences in attention (*p* = 0.004) and verbal memory (*p* = 0.009), were found at the 6-month follow-up.
Hassler et al. 2010	OS	Glioma (WHO grade III–IV)	11	median: 50	IG: holistic mnemonic training	T0: baseline T1: end of course (12weeks)	TMTA; TMTB; HVLT; COWA	Improvement across all neurocognitive variables. Separate dependent t-tests detected no statistically significant group differences for neurocognitive variables (*p* < 0.05) except for HVLT total learning (*p* < 0.05).

OS, observational study; RCT, randomized controlled trial; WHO, world health organization; SD, standard deviation; mo, months; IG, interventional group; CG, control group; PT, physical therapy; OT, occupational therapy; FIM, functional indipendence measure; FMA, fugl-meyer assessment; BBS, berg balance scale; MBI, modified barthel index; MMSE, mini-mental state examination; IQ, intelligence quotient; ECOG, Eastern cooperative oncology group; 10Mwt, 10-meter walking test; MGHFAC, massachusetts general hospital functional ambulation classification; EORTC-QLQ-30, European organization for research and treatment of cancer questionnaire; GHS/QoL, global health status/quality of life; FAB, frontal assessment battery; TMTA and TMTB, trail making tests A and B; HVLT, hopkins verbal learning test; COWA, controlled oral word association test; TEA, test of everyday attention.

**Table 2:** Rehabilitation intervention description.

Reference	Protocol “What procedures?”	Duration; Number of Sessions	Setting "Where?"	Time "When?"
Physical Rehabilitation				
Hansen et al. 2017	(1) 5–10 min of initial warm-up (2) cardio-training of 20 min of cycling or treadmill with intensities ranging from 65% to 85% of the heart rate reserve monitored by pulse (Polar FT40), (3) strength training of main extremity muscle groups with increasing load ranging from 15 to 10 repetition maximum (leg press, arm flexion and arm extension, knee flexion and knee extension), (4) body awareness training or relaxation tailored to personal needs. OT: individual interventions of, offered only to patients who subjectively experienced deficits in occupational performance as evaluated by the COPM	PT: 1 h 30 min/day 3 days/week 18 sessions OT: 1 h/day 2 days/week 12 sessions	Outpatient	na
Ghering et al. 2018	Individualized exercise prescription, based on level of aerobic fitness, to exercise at 60%–85% of maximum heart rate. Patients could choose one or more central activities. The physiotherapist instructed patients in how to use a sports watch with a heart rate monitor, and how to upload training data at least once a week. During the activities, this watch provided immediate feedback about heart rate and, if applicable, speed and distance.	3 days/week 6 weeks 18 sessions	Home	>6 mo After surgery, RT and CT
Yu et al. 2019	PT: range-of-motion exercise, progressive resistive exercise, balance training, endurance training, gait training. OT: cognitive ability enhancement, upper extremity proprioceptive neuromuscular facilitation, and activities of daily living (ADL) training.	PT: 1 h/day OT: 1 h/day 5 days/week	Inpatient	<4 mo since surgery
Hansen et al. 2020	PT: Cardiovascular intensities of up to 75% of the heart rate reserve and resistance training of 3 continuous series with loads progressing from 70% (12 repetitions) to 75% (10 repetitions) of the 1-repetition maximum. 15 min of individual physical therapy for each participant's specific deficits or impairments were allocated. OT: tailored activities based on a screening tool for occupational therapy.	PT: 1 h 30 min/day 3 days/week 18 sessions OT: 1 h/day 2 days/week 12 sessions	Inpatient	na
Tay et al. 2022	PT: passive/assisted stretching exercises, endurance training, strengthening and balance and gait training. OT: cognitive rehabilitation and activities of daily living. Psychological interventions provided counselling and support as required.	3 h/day 5 days/week	Inpatient	After surgery, RT and CT
Cognitive Rehabilitation				
Gehring et al. 2009	The intervention included cognitive retraining and compensation training. The retraining component used a computer program (C-Car37) with hierarchically graded tasks to strengthen attention, as attention deficits are commonly experienced by glioma patients. The compensation training component had six sessions with didactic and practical elements to help patients compensate for impaired cognitive functions. Homework assignments were also given to help with retraining and applying compensatory strategies in daily life.	2 h/session 1 session/week 6 sessions	Outpatient	>6 mo After surgery, RT and CT
Hassler et al. 2010	Dr Stengel's holistic mnemonic training: focus on using all senses, emotions, and intellect to boost memory. Each session addresses perception, concentration, attention, memory, retentiveness, verbal memory, and creativity. Particular attention is paid to concentration, and short-term memory is strengthened to enable learning new information and providing mnemonics. Exercises are designed to help with everyday life. The purpose is to enhance mental capacity, activate restricted abilities, and improve social skills.	1 h 30 min/session	Outpatient	After surgery, RT and CT

PT, physical therapy; OT, occupational therapy; h, hour; CT, chemotherapy; RT, radiotherapy; na, not available.

### 2.2. Physical therapy

Motor deficits associated with brain tumors can vary greatly and include focal deficits such as hemiparesis/plegia with spasticity, gait impairment, ataxia, and incoordination. These impairments depend on the location and extent of the tumor or its treatment, and may affect different muscles or limbs (12). Patients may also experience muscle weakness, fatigue, and reduced physical activity due to chronic steroid use and inflammation caused by the tumor. Furthermore, patients may have trouble performing complex movements, lose motor skills, and become clumsy. These motor impairments can impair mobility and daily functioning, and can result in pain, anxiety and/or depression, loss of independence, and lower quality of life. Therefore, patients need appropriate treatment to improve their motor function, prevent complications of immobility such as pressure ulcers, muscle atrophy, and contractures, and enhance their well-being and quality of life (13). Rehabilitation aims to address the causes of motor dysfunction and the effects it has on physical function, well-being, and quality of life (14).

Five studies were included: two RCTs and three observational studies that examined inpatient, outpatient, and home-based rehabilitation for benign and malignant brain tumors. Different grades of brain tumors were included in all studies. The timing of the rehabilitation varied from study to study—from early after surgery to weeks after the completion of proper management. Rehabilitation protocols usually include physical therapy, occupational therapy, psychological support, and cognitive therapies.

Yu et al. (15) in a retrospective study on the effectiveness of intensive rehabilitation for people recovering from a brain tumor surgery, found that intensive rehabilitation during hospitalization (2 h/die, 5 days/week) after brain tumor surgery resulted in a significant improvement in the outcomes of motor, cognition, and ADL function regardless of the type of tumor. The program included range-of-motion exercise, progressive resistive exercise, balance training, endurance training and gait training.

Tay et al. (16) provided evidence for the effects of an inpatient protocol including passive/assisted stretching exercises, endurance training, strengthening, balance and gait training. They found an improvement in the FIM score, describing a mean gain in motor FIM of 18.8 (SD = 12.9) and a mean gain in cognitive FIM of 3.85 (SD = 6.31) from admission to discharge.

Hansen et al. (17) conducted an interdisciplinary outpatient rehabilitation intervention to assess safety and feasibility. The intervention was a 90-minute protocol, three times a week, with up to four patients in a group exercising together, including warm-up exercise, cardio-training, strength training, and body awareness training or relaxation tailored to personal needs. The results were impressive as patients experienced improved HRQOL and symptom outcomes in terms of EORTC-QLQ-30 “emotional function”, symptom item “fatigue”, and EORTC-QLQ-BN20 symptom scale “future uncertainty”. Furthermore, participants increased their muscle strength and reduced the step frequency for walking 10 meters. Moreover, participants were satisfied with the results. Some years later, the same authors (18) another study to see how effective a 6-week therapy program would be. This program included both physical and occupational therapy with a strong focus on exercise. The exercise included cardiovascular training, resistance training and personalized interventions to help with each participant's specific needs. The results of this trial indicated that the participants who received the intervention did not experience an overall improvement in quality of life, compared to those who received usual care during active anticancer treatment. However, they did display greater aerobic power and muscle strength compared to the control group. Six weeks might be not enough time to observe an impact on overall quality of life, as well as the choice of overall quality of life as the primary outcome measure.

Gehring et al. (19) recently conducted a successful pilot RCT exploring the feasibility of a home-based aerobic exercise intervention for people with grade II and III gliomas. The intervention included three aerobic training sessions per week for six months, and a physiotherapist provided individualized exercise prescriptions based on each patient's aerobic fitness. With the help of a sports watch with a heart rate monitor, patients monitored their heart rate and other metrics during their activities, while the physiotherapist regularly monitored the training data. This program featured several features to ensure adherence, such as allowing patients to exercise at home and providing regular guidance from technology and a physiotherapist. The results showed that the intervention was effective in improving physical fitness, with no adverse events reported. Moreover, cognitive test performance showed trends towards better cognitive performance in the exercise group, with differences in favor of the exercise group observed on measures of attentional inhibition, attention span, auditory selective attention, and working memory (20).

### 2.3. Cognitive therapy

Cognitive dysfunctions are common and debilitating consequences of brain tumors that affect patients' quality of life and daily functioning. The causes of these impairments are complex and depend on tumor characteristics, such as type, location, size, and edema, as well as on the involvement of neural pathways. The most frequently affected cognitive domains are attention (30%) and working memory (20%), followed by visual memory (14%), verbal memory (13%), information processing speed (11%), and executive functioning (8%) (21). The prevalence of cognitive impairment in low-grade glioma varies widely, ranging from 31%–75% before treatment, with higher rates for tumors in the dominant hemisphere (22). Cognitive recovery usually occurs within 3–6 months, but 19%–83% of patients remain impaired (23). Low-grade patients commonly demonstrate cognitive impairments in attention and concentration, processing speed, learning and memory, and executive functions (24). High-grade tumors can cause brain tissue to atrophy, leading to slower or stopped plasticity processes and more disabling deficits in cognitive domains (25). Cognitive rehabilitation interventions can help patients with brain tumors cope with their cognitive impairments and improve their quality of life. Previous studies have shown promising outcomes and high satisfaction levels among patients and their families who received these interventions (26).

This mini-review included two studies with a focus on cognitive rehabilitation, one randomized control trials and one observational study.

Gehring et al. (27) explored the impact of a cognitive rehabilitation program (CRP) on objective and subjective measures of cognitive ability in patients with gliomas in remission. The program consisted of six weekly 2-hour sessions and incorporated both cognitive retraining and compensation training, with a focus on attention in the retraining component and attention, memory, and executive functions in the compensation component. Results showed that self-reported cognitive functioning improved after the intervention and there were distinct differences in attention and verbal memory observed at the 6-month follow-up. The intervention also had a significant effect on long-term mental fatigue scores. However, there was no significant difference between the two groups for self-reported mental health-related quality of life or community integration. Both groups showed improved objective cognitive performance in the short term, but the CRP group demonstrated continued improvement at 6-month follow-up, while the control group did not.

Hassler et al. (28) in a pilot study found that over a 3-month period, patients with glioblastoma improved their attention and memory skills after participating in weekly group training sessions. This training discipline uses exercises to train perception, concentration, attention, memory, retentiveness, verbal memory, and creativity Its goal is to improve cognitive function, maintain functional potential for intellectual and memory abilities, and reactivate any limited capacities. The training environment is stress-free and in a small group setting to also foster social skills development. Special emphasis is placed on training concentration skills and empowering short-term memory, and mnemotechnics are used to help attendees learn new information. Neuropsychological tests revealed improvements in all cognitive functions on a group basis, but individual patients showed both improvement and deterioration in cognitive performance.

## 3. Discussion

The studies reviewed explore protocols of various rehabilitation interventions for glioma patients. Rehabilitation protocols typically include physical therapy, occupational therapy, psychological support, and cognitive therapies, and the timing of the rehabilitation can vary from early after surgery to weeks after the completion of proper management. The interventions included inpatient, outpatient, and home-based rehabilitation, with varying exercises and therapy components.The studies showed that rehabilitation can lead to improvements in motor function, cognition, activities of daily living, and quality of life, with some studies also demonstrating improvements in physical fitness and cognitive performance. However, it is important to note that the timing, intensity, and duration of rehabilitation may influence its effectiveness. Some studies showed mixed results, highlighting the need for further research in this area. Overall, rehabilitation interventions can play an important role in improving the outcomes and well-being of patients with brain tumors. It is important for healthcare providers to consider rehabilitation as part of the comprehensive care for these patients and to tailor the interventions to individual needs and circumstances. Infact, the interventions with the most successful outcomes were those that included personalized exercise prescriptions, individualized training, and adherence strategies such as monitoring training data and regular guidance from a physiotherapist.

As for the cognitive rehabilitation protocol, the two studies included in this mini-review provide valuable insights into the effectiveness of cognitive rehabilitation interventions for patients with gliomas. Gehring et al. highlights the potential benefits of early cognitive rehabilitation interventions for patients with gliomas in remission; while Hassler et al.'s pilot study also showed promising results, indicating that group training sessions targeting attention and memory skills can lead to improvements in cognitive function, particularly in perception, concentration, and retentiveness. Overall, these studies suggest that cognitive rehabilitation interventions can significantly contribute to improvement in the quality of life of patients with gliomas and should be further explored in larger and more rigorous studies.

It is noteworthy that some studies were excluded because they included other patients besides gliomas. However, they are still relevant for glioma rehabilitation and may offer useful insights. So, we summarized their findings in the discussion section and provided detailed information about their protocols in the Supplementary Material.

Regarding the physical rehabilitation a study by Yoon et al. (29) investigated the potential of combining VR-based rehabilitation with conventional Occupational Therapy (OT) to improve upper extremity function in patients with brain tumors, including glioma. They found that the intervention group showed more improvement in proximal UE function, while the control group experienced better fine motor function and overall coordination. Bartolo et al. (30) also found significant improvements in motor outcomes with a physical therapy intervention after surgical treatment, which included patients with glioma. Beginning soon after surgical treatment, the physical therapy intervention consisted of one hour per day, six days a week, and a protocol of five phases, each lasting 10 to 15 min. These phases passive and active stretch and strengthening exercises, balance, and walking training.

Concerning cognitive rehabilitation, we discovered five studies that incorporated glioma patients as well as those with other brain tumor types. For instance, Zucchella et al. (31) demonstrated the efficacy of cognitive training in post-surgical patients, which suggests that early intervention may be beneficial for improving cognitive function in brain tumor patients. A 16-session program combining direct training and metacognitive training was conducted by psychologists to enhance cognitive abilities. Patients completed computer exercises targeting various cognitive functions and were provided guidance on tasks and metacognitive strategies. Significant improvement in visual attention and verbal memory was observed, suggesting a successful retraining approach to improving cognitive performance. Other cognitive domains may also benefit from improvements in attention. Yang et al. (32) showed that combining virtual reality with computer-assisted cognitive rehabilitation can have positive effects on cognitive improvement in brain tumor patients. Maschio et al. (33) found that cognitive rehabilitation training can improve short-term verbal memory, episodic memory, fluency, and long-term visuospatial memory in patients with brain tumor-related epilepsy and cognitive issues. The cognitive rehabilitation training consisted of 10 weekly individual 1-hour sessions, each led by a trained psychologist, and focused on memory, attention, visuo-spatial functions, language, and reasoning. Richard et al. (34) demonstrated that Goal Management Training, a behavioral intervention combining mindfulness and strategy training, could improve executive functions and functional goal attainment in brain tumours patients. Finally, Van der Linden et al. (35) showed that a tablet-based cognitive rehabilitation program can be effective in improving cognitive performance, cognitive complaints, fatigue, and psychological distress in primary brain tumour patients following neurosurgery.

## 4. Conclusion

This review shows that rehabilitation interventions, such as physical and cognitive therapies, can enhance quality of life and function in glioma patients. Rehabilitation is a safe, feasible, and well-tolerated option that should be considered for glioma patients. However, more studies are required to clarify the rehabilitation effects on glioma patients, such as finding the best rehabilitation models, assessing motor and cognitive outcomes, choosing the appropriate physical activity strategies, evaluating the long-term training effects, and measuring the influence of motor and cognitive rehabilitation on patient daily life and caregivers. New approaches such as neuromodulation have been proposed as a new strategy to increase the safety of brain tumor resection, promote cortical reorganization and accelerate functional recovery after surgery (36, 37). Investigating the functional connectivity of gliomas to healthy brain regions could help to accurately assess tumor aggressiveness and inform therapeutic and rehabilitation strategies (38, 39). A multidisciplinary approach and powerful collaboration among healthcare professionals, with extensive experience in the area, are essential (40). Future clinical trials are needed to optimize multimodal interventions to mitigate the glioma consequences and provide optimal care to patients.

## Author contributions

SS, SF: concept and design. SS: drafting and reviewing the manuscript. SF: literature analysis and manuscript revision. NC, RP, PF and AS: technical support and study supervision. All authors contributed to the article and approved the submitted version.

## Conflict of interest

The authors declare that the research was conducted in the absence of any commercial or financial relationships that could be construed as a potential conflict of interest.

## Supplementary material

The Supplementary Material for this article can be found online at: https://www.frontiersin.org/articles/10.3389/fsurg.2023.1137516/full#supplementary-material.


