# Supplementary material for: Rehabilitation interventions for glioma patients: a mini-review
Source: Front Surg. 2023 Jun 16;10:1137516. doi: 10.3389/fsurg.2023.1137516 (PMC10313351; doi:10.3389/fsurg.2023.1137516)
Supplement: Supplementary file 1 [file Datasheet1.docx]

SUPPLEMETANTARY MATERIAL

**Figure S1 : Prisma Flow Chart**

**
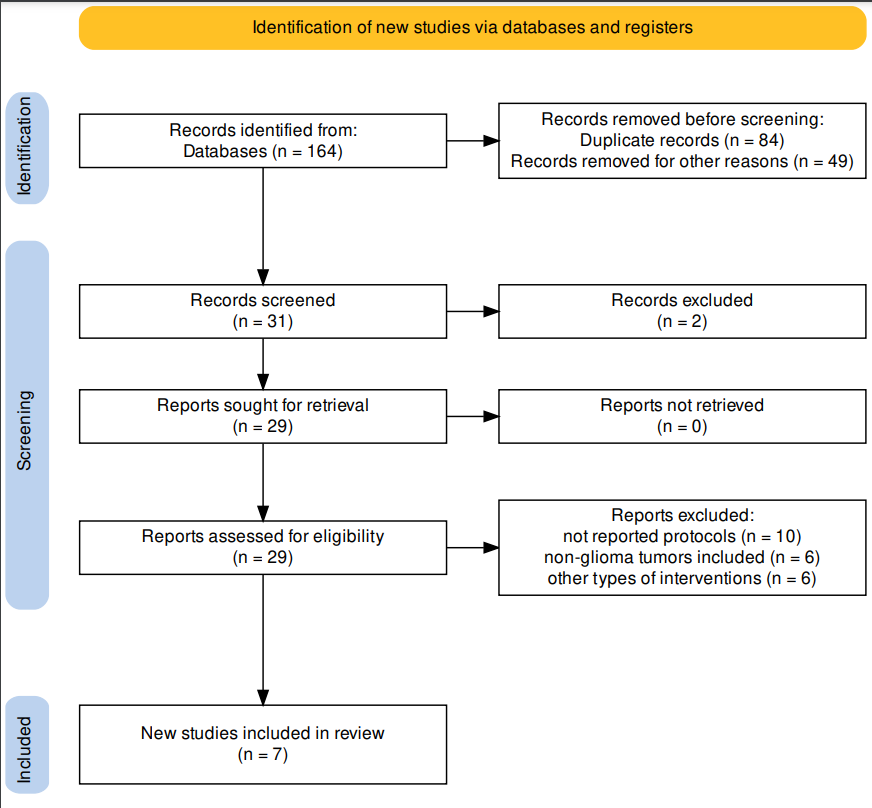
**

**Table SI: Characteristics of the included studies.**

| Reference | Study design | Diagnosis | Sample size | Age (years) mean±SD | Rehabilitative Intervention | Evaluation timing | Outcomes | Main Results |
| --- | --- | --- | --- | --- | --- | --- | --- | --- |
| *Physical Rehabilitation* | | |  |  |  |  |  |  |
| Bartolo et al. 2012 | OS | Glioma (WHO grade IV), MEN | 75 (43 MEN; 32 GB) | 63.5 ± 13.2 | IG: PT | T0: baseline T1: end of treatment (4 weeks) | Sitting Balance; Standing Balance; Hauser Index; MGHFAC; FIM | Significant improvement in all the outcomes (p < 0.001). |
| Yoon et al. 2015 | OS | Glioma (WHO grade I-IV), MEN; MET | 40 (20 IG; 20 CG) | IG: 48.6 ± 11.3 CG: 50.0 ± 17.5 | IG: Virtual reality training  CG: OT | T0: baseline  T1: end of treatment (3 weeks) | Box and Block test; Manual Function test; FMA; MBI | Statistically significant improvement in the BBT, MFT, FMS in the IG. By contrast, the CG showed significant improvement in hand dexterity (FMS) and hand subsection (FMS). |
| *Cognitive Rehabilitation* | | |  |  |  |  |  |  |
| Zucchella et al. 2013 | RCT | Glioma (WHO grade I-IV), MEN and ohers | 53 (25 IG; 28 CG) | IG: 58.7±13.9;  CG: 52.7 ±17 | IG: computer exercises + metacognitive training CG: standard care | T0: before treatment T1: end of treatment (4 weeks) | MMSE; Digit Span; Corsi’s Test; RAVLT; logical memory  immediate and delayed recall; PM47; FAB; TMTA; TMTB; Attentive Matrices; ROCF; Verbal fluency | The IG performed better than the control group in most of the neuropsychological tests. However, a statistically significant difference was found only in the domains of visual attention and verbal memory. |
| Yang et al. 2014 | RCT | Glioma (WHO grade III-IV), MEN and others | 38  (19 IG; 19 GC) | IG: 47.9±14.5;  CG:  52.9 ± 14.0 | IG: Virtual reality training + CBCT CG: CBCT | T0: baseline T1: end of treatment (4 weeks) | VCPT; ACPT; WCW; FDST; BDST; FVST; BVST; ViLT-R; VeLT-R; TMT-A; MBI; MMSE | Statistically significant (p < 0.05) improvements in the visual and auditory CPTs, FDST, BDST, FVST, BVSTs, verbal and visual learning tests, and TMT-A in the IG group. In the control group after the treatment, statistically significant (p<0.05) improvements were observed in the ACPT, ViLT-R VeLT-R, and FVST. Both MMSE and MBI showed improvements in both groups after the treatment. |
| Maschio et al. 2015 | OS | Glioma (WHO grade I-IV), MEN; MET | 12 | 48.6. Interval 28-68 | IG: conventional Cognitive training | T0: before treatment T1: end of treatment T2: 6 mo | MMSE; TMT; FAB; Raven progressive Matrices; ROCF; Word List, delayed recall and story recall; Clock Drawing test; Span forward and backward; Recall of disyllabic words; semantic fluency tests | Significant improvement in span forward (p = 0.017), ROCF-recall (p = 0.036), story recall (p = 0.036) and phonetic fluency test (p = 0.043). |
| Richard et al. 2019 | RCT | Glioma (WHO grade I-IV), MEN and ohers | 26 (11 IG; 8 ACG; 6 CG) | 47.7 ±:11.5 | IG: Cognitive training; ACG: psycho-educational interventions; CG: standard care | T0: baseline T1: end of treatment T2: 4 mo later | TMTA; TMTB; TEA; SART; BADS; Zoo Map Test; HVLT-R; BRIEF-A; FrSBe; PANAS; HADS; IIRS | Executive functions improved with IG but not ACG or CG (post-training p = 0.077, follow-up p = 0.046). Functional goal attainment was greatest with IG (post-training p = 0.027, follow-up p = 0.064). |
| Van der Linden et al. 2021 | RCT | Glioma (WHO grade II-III), MEN | 62 (31 IG; 31 CG) | IG: 45.7±11.7;  CG: 52.6±10.4 | IG: Tablet-based cognitive rehabilitation CG: waiting list | T0: before surgery T1: 3 mo after surgery T2: 6 mo after surgery T3: 12 mo after surgery | Neuropsychological test battery Central Nervous System Vital Signs; Digit Span Test; Letter Fluency; CFQ; BRIEF-A; MFI-20; HADS | Significant positive main effects of time (irrespective of group) were observed for processing speed (p < 0.001), complex attention (p = 0.003), cognitive flexibility (p < 0.001) and working memory (p = 0.048). |

OS: Observational Study; RCT: randomized Controlled Trial; WHO: World Health organization; SD: Standard Deviation; mo: months; MET: metastasis; MEN: meningioma; IG: Interventional Group; CG: Control Group; PT: Physical Therapy; OT: Occupational therapy; FIM: Functional Indipendence measure; FMA: Fugl-Meyer Assessment; BBS: Berg Balance Scale; MBI: Modified Barthel Index; MMSE: Mini-Mental State Examination; IQ: Intelligence quotient; ECOG: Eastern Cooperative Oncology Group; 10Mwt: 10-meter walking test; MGHFAC: Massachusetts General Hospital Functional Ambulation Classification; EORTC-QLQ-30: European Organization for Research and Treatment of Cancer Questionnaire; GHS/QoL: global health status/quality of life; FAB: Frontal Assessment Battery; TMTA and TMTB: Trail Making Tests A and B; HVLT: Hopkins Verbal Learning Test; COWA: Controlled Oral Word Association Test; BRIEF-A: Behavior Rating Inventory of Executive Function-Adult Version; TEA: Test of Everyday Attention; SART: Sustained Attention to Response Tas; BADS: Behavioural Assessment of the Dysexecutive Syndrome; HVLT-R: Hopkins Test of Verbal Memory-Revised; FrSBe: Frontal Systems Behavior Scale; PANAS: Positive and Negative Affect Schedule ; HADS: Hospital Anxiety and Depression Scale; IRSS: Illness Intrusiveness Rating Scale; MFI-20: Multidimensional Fatigue Inventory; CFQ: The Cognitive Failures Questionnaire; ROCF: Rey-Osterrieth Complex figure; PM47: Raven’s Coloured Progressive Matrices 47; CBCT: computer-based cognitive training; VCPT: Visual continuous performance test; ACPT: Auditory continuous performance test; WCW: Word of color word in word-color test; FDST: Forward digit span test; BDST: Backward digit span test; FVST: Forward and Backward visual span test; BVST: Backward visual span test; ViLT-R: Visual learning test-recognition; VeLT-R: Verbal learning test-recognition

**Table SII: Rehabilitation intervention description**

| Reference | Protocol “What procedures?" | Duration; Number of Sessions | Setting "Where?" | Time  "When?" |
| --- | --- | --- | --- | --- |
| *Physical Rehabilitation* |  |  |  |  |
| Bartolo et al. 2012 | 1) Passive/assisted stretching exercises for the shoulder,elbow, ankle, and knee (10 min) 2) Passive/assisted strengthening exercises for trunk muscles and upper and lower limbs (10 min); 3) Strengthening exercises in a functional context to promote upper limb and/or hand use (15 min); 4) Balance exercises, initially in sitting position and then in standing position (once the patient had recovered the ability to stand, even with assistance) (10 min); 5) Ground-floor walking (including step control) first with assistance, then under supervision (15 min). | 1h/day 6 days/week | Inpatient | 2 weeks after surgery |
| Yoon et al. 2015 | Conventional OT: range of motion exercises, fine motor training, and strengthening UE exercises.Range of motion exercises consisted of overhead pulley, range of motion arc, and inserting rings to a horizontal bar. Fine motor training consisted of turning coins,tapping of the thumb and the index finger, as well as inserting a piece of wood into small holes in a pegboard. Strengthening exercises consisted of upper body exerciser, sanding, weight pulley exercises, and using a Theraband or lifting a dumbbell. Each exercise took approximately 10 mins. Virtual Reality: Interactive Rehabilitation and Exercise (IREX) System. Six VR exercises (Birds and Balls, Conveyor, Drums, Juggler, Coconuts, and Soccer) | PT: 30 mins / day, 3days/week  9 sessions  OT: 30 mins / day, 2days/week  6 sessions | Inpatient | na |
| *Cognitive Rehabilitation* |  |  |  |  |
| Zucchella et al. 2013 | Combining computer exercises (remedial approach) therapist- guided computer exercises with increasing difficulty levels, targeting different cognitive functions: time orientation, spatial orientation, visual attention, logical reasoning, memory, executive function. Metacognitive training (compensatory approach): strategies to increase awareness, foster motivation and promote self-regulation. | 45 min/session  4 sessions/week  16 sessions | Inpatient | 2 weeks after surgery |
| Yang et al. 2014 | Computer based training: ComCog (MaxMedica Inc., Seoul, Korea) was used for computer-assisted cognitive rehabilitation. This system contained programs designed to enhance attention and memory skills, with tasks varying in difficulty based on patient condition. Attention programs included attention training, discrimination training, perception training, and emotion training. Memory programs included spatial memory training, recognition memory training, recall memory training, and categorization memory training.  VR training: Interactive Rehabilitation and Exercise (IREX) System. Six VR exercises (Birds and Balls, Conveyor, Drums, Juggler, Coconuts, and Soccer) were used. | VR training: 30 minutes/die 12 sessions  CR: 30 minutes/die 2 times a week 8 sessions | Inpatient | na |
| Maschio et al. 2015 | A multimedial software called TNP (Training Neuropsicologico) that allows the treatment of almost all cognitive deficits from focal lesions. The functions trained were: memory, attention, visuo-spatial functions, language and reasoning. Memory training focused on short term and long term verbal, and visuo-spatial memory using word lists or images. Attention training stimulated alternating, selective, divided and sustained attention. | 1h/session 1 session/ week 10 sessions | Outpatient | After surgery (years) |
| Richard et al. 2019 | A behavioral intervention developed from neurocognitive models of executive functioning called Goal Managment Training (GMT). It addresses executive and related attention, memory, and behavioral impairments. GMT homework involved monitoring cognitive problems and applying learned strategies to day-to-day activities. | 2h/session 1 session/ week 8 sessions | Outpatient | ≥ 3 mo post-radiation or surgery |
| Van der Linden et al. 2021 | A tablet-based cognitive rehabilitation program ReMind includes psychoeducation, strategy training and an attention retraining game. The psychoeducational information and strategy training are spread over six modules, namely (1) Cognitive functions, (2) Influences, (3) Compensation, (4) Attention, (5) Planning & Control, and (6) Memory. | 3h/week  10 weeks | Home | 3 mo afters surgery |

PT: Physical Therapy; OT: Occupational therapy; h: hour; CT: chemotherapy; RT: radiotherapy; na: not available
